# Sustaining Situational Assessment in Electrical Network Power Control

**DOI:** 10.1177/00187208241272072

**Published:** 2024-08-06

**Authors:** Mark W. Wiggins, Jaime C. Auton, Daniel Sturman, Ben W. Morrison, Brett R. C. Molesworth

**Affiliations:** 1 7788 Macquarie University, Australia; 2 1066 University of Adelaide, Australia; 3 7800 University of New South Wales, Australia

**Keywords:** electricity, network control, situational assessment, cue utilisation

## Abstract

**Objective:**

The aim of this study was to examine contributions to sustained situational assessment over an extended period in the context of electricity transmission control.

**Background:**

The electricity industry is engaged in a period of unprecedented change in the transition to renewable sources of energy. Changes in the nature and function of electricity transmission risks a reduction in situational assessment as network controllers place increased reliance on advanced technology to identify and diagnose changes in the system state.

**Method:**

Transmission network controllers from three organisations completed an assessment of their situational assessment on two occasions, one year apart.

**Results:**

Multiple regression revealed a statistically significant model in which the variance in Year 2 was predicted by a combination of performance in Year 1, the recency of formal training, and the extent to which controllers perceived their job as exciting. No relationship was evident for years of experience as a network controller.

**Conclusion:**

The results suggest that a combination of recent formal training and perceptions of job excitement may have implications in maintaining the capacity for situational assessment over an extended period in the context of electricity network control.

**Application:**

The outcomes of the present study suggest that changes in situational assessment can be monitored and that strategies, including formal training and job design, may sustain situational assessment over an extended period in advanced technology settings.

## Introduction

Effective and efficient control of an electrical system is complex and demands the capacity to identify changes in the system state and diagnose the nature of these changes in preparation for a response. Referred to as situational assessment, this process involves deriving relationships between environmental features and aggregating interpretations of object states to form an understanding of the state of a system (Blasch et al., 2006). This determines the need for intervention to balance the generation of electricity against demand.

Like other industrial environments, the electricity industry is engaged in a period of change, the most significant of which is the transition to renewables (Sajadi et al., 2019). This transition has implications for the operation of the network since it has the potential to create fluctuations in electrical voltage and frequency that require an additional level of situational assessment. This oversight is necessary to ensure that optimal levels of service are maintained for customers.

Alongside changes to the generation and transmission of new sources of energy, new technologies are being introduced that are intended to provide additional support for electricity controllers. This involves increasing the use of automation for situational assessment, thereby reducing the demands on cognitive resources. It also allows the centralisation of situational assessment through closed-loop systems that operate in the absence of controller involvement (Sayed & Gabbar, 2017).

While the application of closed-loop systems enables rapid, reliable responses to changes in the network, human controllers remain necessary in an oversight role should the automated systems fail to respond when and/or where necessary. Over extended periods, this role of oversight can induce boredom and a lack of job excitement, the outcome of which is a detachment or disengagement from job tasks (Cummings et al., 2013, 2016). This limits the opportunity to develop and reinforce the skills necessary to maintain the capacity for situational assessment.

### Situational Assessment

Accurate and efficient situational assessment draws on preexisting associations in long-term memory (Fracker, 1988). Represented as schemas, productions, or cues, it comprises associations between environmental stimuli and events or objects (Klein et al., 1989). These associations are acquired through statistical learning and the coexistence of features and events or objects that infers a relationship (Van den Bos et al., 2012). Over frequent trials, the perceived relationship is strengthened to the point where the presence of the environmental stimuli activates the association in the absence of conscious awareness, providing the initial basis for situational awareness (Endsley & Garland, 2000).

Activations of memory-based associations in the absence of conscious awareness relieves the demands on information processing and short-term memory, and increases the rate at which accurate and comprehensive situational assessment can be achieved (Mayer & Moreno, 2003). This has advantages in high consequence, time-constrained environments, where delays in situational assessment can lead to rapid deteriorations in the system state, potentially impeding recovery efforts. In the context of electricity control, delays can also result in the destruction of infrastructure and, in some cases, loss of life.

Opportunities to develop associations in memory occur through interactions with the operational context, normally through targeted training and/or on-the-job exposure. However, individual differences in perceptions of the job, together with the idiosyncratic nature of operational interactions, tends to result in differences between operators in both the rate at which associations are acquired and the nature of the associations that are acquired. Together with individual differences in the inherent capacity to draw associations in memory, assuming the capacity for situational assessment simply as a function of exposure tends to be problematic.

Assessments of situational assessment are generally directed towards accurate interpretations of problem-oriented scenarios. For example, in the medical context, the capacity for situational assessment has been inferred from the frequency of accurate diagnoses, having been presented a series of symptoms (Porat et al., 2016). Similarly, the accuracy of a pilot’s mental representation at various stages of a scenario is assumed indicative of the capacity to comprehend the system state (Wiegmann et al., 2002).

While high fidelity, scenario-based evaluations are useful assessments, they can be costly to design and implement, particularly in technology-oriented settings. Comparisons of performance can also be problematic since different operators may select different paths towards problem resolution, some of which may be more appropriate than others under the circumstances. Finally, since scenario-based evaluations take time, operators may only experience a limited number of scenarios, constraining the generalisability of the outcomes and a broader assessment of inherent capabilities, including the availability of a broad range of cues.

The alternative to high fidelity scenarios is an approach that targets the inherent capabilities that enable successful cognitive processing. For example, if effective and efficient situational assessment depends on a repertoire of cues in memory, then establishing the availability of these cues should correspond to successful performance. The challenge lies in the idiosyncratic and unobservable nature of cues so that their existence must be inferred based on performance in response to stimuli that would be expected to invoke cue-oriented responses.

In response to a stimulus, where cues are available in memory, performance would be expected to be rapid and accurate. Further, in response to an event, critical cues would be expected to be prioritised during information acquisition and the strength of association between related stimuli should be readily apparent. Finally, during problem-solving, relevant cues should discriminate more from less relevant cues. In combination, these behavioural responses constitute different strategies to assess cue utilisation (Wiggins, 2021).

Framed as a comprehensive approach to the assessment of cue utilisation, it offers a proxy measure for the capacity for situational assessment. Patterns of behaviour in response to task-related stimuli infer the application of cue-based associations from memory. This approach accounts for individual differences in the repertoire of cues in memory and the different patterns that might be applied in the interpretation of a scene, despite equivalent levels of accuracy and response latency in situational assessment.

Behavioural assessments of cue utilisation have been employed to assess situational assessment across a range of domains, including paediatric intensive care (Loveday, Wiggins, Searle et al., 2013), radiology (Carrigan et al., 2021), software engineering (Loveday et al., 2014), and network power control (Loveday, Wiggins, & Harris et al., 2013). Assessments of the validity of the approach have included peer-assessments of expertise (Loveday, Wiggins, Harris, et al., 2013; Loveday, Wiggins, Searle, et al., 2013), performance on a simulated diagnostic task (Carrigan et al., 2021), performance in practice (Loveday et al., 2014), eye tracking behaviour (Sturman & Wiggins, 2021), and neural activity (Sturman et al., 2019). The test-retest reliability of these assessments has been demonstrated in both short and extended-exposure learning tasks.

### EXPERTise 2.0

In network power control, differences in cue utilisation have been evaluated using the EXPERT Intensive Skills Evaluation (EXPERTise) 2.0 online assessment tool that incorporates tasks that are intended to offer different approaches to the assessment of domain-related cue utilisation (Small et al., 2014). Differences in performance across the tasks corresponded to differences in subjective assessments, where higher cue utilisation was associated with greater experience in network power control. Similarly, higher cue utilisation is associated with lower cerebral blood oxygenation in the prefrontal cortex amongst network power controllers in practice (Sturman et al., 2019). In combination, this suggests that greater levels of exposure to the operational context is associated with the opportunity to acquire cues, resulting in lower levels of cognitive demand during operational activities.

While the relationship between cue utilisation and operational performance is reasonably well-established, it remains unclear how cue utilisation is maintained over time. This is particularly true in an organisational context where there are likely to be individual differences in opportunities for structured training and development. Similarly, there are likely to be differences in on-the-job exposure that contribute to cognitive development. Parker (2014) suggests that cognitive development is distinct from the cognitive change that arises from structured learning and training.

Jobs that are perceived as more exciting tend to involve less routine cognitive strategies, demanding higher-order cognitive processes that initiate cognitive development (Richters et al., 2016). With on-the-job exposure, the organisation of knowledge is altered through feedback and there is an opportunity to establish or consolidate rule-based relationships. These relationships constitute new or more precise associations in memory, thereby contributing to cue utilisation.

New or precise associations are necessary when new technologies are introduced that alter the features through which operators interact with the operational environment. For example, the management of electricity networks is continuously evolving with the development of new capabilities for automation, changes to human–machine interfaces, and the application of more capable decision support systems. The consequence is a reduction in the number of operators necessary to oversee the network, but an increasing reliance on those operators to maintain their skills and capabilities necessary to ensure a timely and accurate response to changes in the system state.

The aim of this study was to test the contribution of overall experience, recency of training, perceptions of job excitement or boredom, and prior performance on cue utilisation in the context of transmission network power control.

### The Present Study

Consistent with previous research, cue utilisation in the present study was assessed using EXPERTise 2.0. The cue utilisation of experienced transmission power controllers was assessed on two occasions, one year apart. In Year 2, participants were asked to indicate their perceived level of excitement that they experience at work, the date of their most recent training, and their years of experience working in network control. The participants constitute a sample drawn from a limited population of approximately 80 highly experienced network controllers from three Transmission Network Service Providers in Australia and New Zealand. It was hypothesised that transmission network controllers’ cue utilisation would be positively related to their overall experience, their previous performance, and their perceived level of excitement and inversely related to the period of time since their most recent training.

## Method

### Participants

The participants comprised 21 qualified electricity transmission network controllers who are responsible for monitoring and diagnosing system events from remote data screens. They were employed in electricity transmission and were operating as transmission network controllers for one of three organisations in Australia or New Zealand.

Overall, the participants were male, aged between 26 and 59 years, and reported a mean 16.14 years (*SD* = 10.27) experience working in power transmission and a mean 10.80 years (*SD* = 7.97) working as a network controller.

### Materials

The participants completed the Transmission Network Power Control (TNSP) edition of EXPERTise 2.0 on two occasions, one year apart. EXPERTise 2.0 is an online platform that comprises tasks that constitute different approaches to the assessment of cue utilisation, together with a flexible survey feature. In completing the survey, participants were asked to indicate their experience (in years) working in power transmission and working as a network controller. They were also asked in Year 2, to indicate, using a six-point Likert-type scale, the extent to which they experienced their role as ‘Boring’ (1) or ‘Exciting’ (6), and whether their last experience of formal training was within the preceding month (1), two months (2), three (3) months, or beyond three months (4), prior to testing at Time 2.

The TNSP edition of EXPERTise 2.0 featured four tasks, the scenarios for which were developed with the assistance of two subject-matter experts with approximately 15 years direct experience in electricity network control. The construct validity of the TNSP edition of EXPERTise 2.0 has been established where cluster analysis based on performance was associated with corresponding levels of diagnostic performance (Loveday, Wiggins, Harris, et al., 2013; Loveday, Wiggins, Searle, et al., 2013).

#### Feature Identification Task

The Feature Identification Task (FIT) is intended to examine participants’ capacity to recognise accurately, a pattern of features following a brief exposure. For the TNSP edition, two types of FIT were included, the first of which comprised a series of 18 Single Line Diagrams (SLD) within which a fault was depicted. Following two practice scenarios, participants were asked to select, as quickly as possible, the area of greatest concern for the safe operation of the power system. In the second FIT, participants responded to a series of 19 lists of alarms following two practice scenarios and were asked to select the open circuit breaker alarm as quickly as possible. Faster response latency in selecting the correct response is associated with a greater capacity for feature identification (Loveday, Wiggins, Harris, et al., 2013; Loveday, Wiggins, Searle, et al., 2013).

#### Feature Association Task

The Feature Association Task (FAT) is designed to examine participants’ capacity to rapidly draw associations between corresponding features. In the TNSP edition, participants reviewed 18 pairs of terms that relate to transmission network control. The terms were presented simultaneously for 500 ms either side of a red cross that was located in centre of screen and to which participants were directed beforehand. Exemplar pairs included ‘voltage depression’ with ‘loss of interconnector’, and ‘load increase’ with ‘lost generation’. Following exposure, participants were presented with a 7-point Likert-type scale ranging from ‘extremely related’ to ‘not at all related’, and both their response and response latency were recorded. A higher mean variance in responses as a proportion of mean response latency is presumed indicative of a greater capacity for feature association (Morrison et al., 2013).

#### Feature Prioritisation Task

The Feature Prioritisation Task (FPT) is intended to capture participants’ prioritisation in their selection of features to resolve a scenario about which minimal information is available initially. For the TNSP edition of EXPERTise 2.0, the stimulus comprised four hypothetical scenarios in which a report had been received such as a particular line had ‘tripped’ or that a bushfire threatened a particular line. Participants were given 60 seconds to source information from a list of between 13 and 15 information tabs to determine their response. If participants completed their review of the information before the 60 second period had elapsed, they selected a button labelled ‘My Decision’ to take them to the following page where they could select their response from a list of four options. If they had not completed their review before the 60 second period had elapsed, they were directed automatically to select one of the four options.

The sequence in which participants accessed information was recorded and segmented into pairs of information tabs. A higher ratio of pairs of information tabs accessed in the sequence in which they were listed, calculated as proportion of the total pairs of information tabs selected during the scenario, is presumed indicative of lower feature prioritisation (Wiggins & O'Hare, 1995).

#### Feature Discrimination Task

The Feature Discrimination Task (FDT) involves the presentation of two detailed scenarios that describe task-related problems. In the TNSP edition, the scenarios comprised a description of details associated with an outage, include the time of day, the location of the outage, and the ambient temperature, together with a single line diagram representing part of the network. Participants were asked to read the information and once they had identified their initial response, select a button labelled ‘My Decision’. Four options were available, and participants selected the option that most closely reflected their intended, initial response.

Having selected their initial response, participants were asked to rate, using 10-point Likert-type Scale, ranging from ‘Not at all Important’ to ‘Extremely Important’, the importance of 19 features described in the scenarios when deciding the initial response. Higher cue utilisation is associated with a greater mean variance across ratings (Pauley et al., 2009).

### Procedure

Following ethics approval from the University Human Research Ethics Committee (52021228326364), participants were recruited from three Transmission Network Control organisations across Australia and New Zealand. Potential participants were invited to complete EXPERTise 2.0 on two occasions, one year apart.

## Results

### Data Reduction and Descriptive Statistics

EXPERTise 2.0 data were calculated by initially aggregating performance across the scenarios that comprised each of four tasks. Consistent with the approach taken in Nasser et al. (2020), these data were standardised subsequently, and a mean (performance score) calculated that constituted overall performance on the Transmission Network edition of EXPERTise 2.0. The unstandardised means for each of the four tasks are listed in Table 1.

**Table 1.** Unstandardised Means and Standard Deviations for the Four EXPERTise 2.0 Tasks in Year 1 and Year 2.

EXPERTise 2.0 Task	Year 1		Year 2	
	Mean	Standard Deviation	Mean	Standard Deviation
Feature identification task (msec)	1842.10	258.58	8248.27	4626.97
Feature association task (response latency/variance)	0.0012	0.00079	0.0005	0.0003
Feature discrimination task (variance)	5.57	2.83	5.07	2.42
Feature prioritisation task (ratio)	0.84	0.12	0.72	0.20

The predictor variables were assessed for normality and checked for potential multicollinearity prior to subsequent analysis. No statistically significant relationships were evident for the Pearson Product-Moment correlations (see Table 2). However, subsequent Bayes JZS correlations revealed what would be regarded by Jeffreys (1998) as substantial evidence for a relationship between performance at Year 1 and training recency and job excitement, and experience and job excitement.

**Table 2.** Correlation Matrix Summarising the Relationship Between the Predictor Variables (Bayes Factors in Parentheses).

Variable	Mean	SD	1	2	3	4
1. Experience (years)	10.84	7.96				
2. Year 1 performance	−.004	0.41	.38 (1.38)			
3. Year 2 recency of training	2.95	1.32	.31 (2.33)	.06 (5.89)		
4. Year 2 perception of job excitement	4.73	0.94	.12 (5.32)	.15 (4.86)	.41 (1.01)	

A hierarchical multiple regression, with years of experience, EXPERTise 2.0 performance in Year 1, recency of formal training, and perceptions of job excitement entered sequentially as predictor variables, was used to determine the combination of variables that predicted performance on EXPERTise 2.0 at Year 2. Entering years of experience explained only 2% of the variance in Year 2 Performance and was nonsignificant, *F(change)* (1, 20) = 0.43, *p(change)* = .521 adj*R*^2^ = −.03. A further 20.9% was explained by EXPERTise 2.0 performance at Year 1, *F(change)* (1, 19) = 2.83, *p(change)* = .04 adj*R*^2^ = .15. This increased by a further 4% with the addition of the recency of formal training, *F(change)* (1, 18) = 0.96, *p(change)* = .34 adj*R*^2^ = .15, and an additional 28.3% with perceptions of job excitement, *F(change)* (1, 17) = 10.7, *p (change)* = .005 adj*R*^2^ = .45

In the absence of direct evidence of multicollinearity (see VIF in Table 3), significant contributions from Year 1 performance, perceptions of job excitement, and the recency of training explained 45% of the variance in performance in Year 2 when all of the predictors were entered (see Table 3). No relationship was evident for years of experience as a network controller.

**Table 3.** Summary of the Regression Model for Performance at Year 2 With all of the Variables Entered.

Predictor Variables	β	B	SE	*t*	Tolerance	VIF
Experience (Years)	.07	0.004	0.01	0.36	0.77	1.29
Performance at year 1	.39	0.43	0.19	2.19*	0.83	1.19
Recency of training	−.46	−0.16	0.06	−2.44*	0.75	1.33
Perceptions of job excitement	.59	0.28	0.08	3.27*	0.81	1.23

*F*(4, 17) = 5.22, *p* = .006 adj*R*^2^ = .45.

*p < .05.

Amongst the participants, the perception of greater job excitement was associated with greater performance on the Transmission Network Power Control edition of EXPERTise 2.0 at Year 2. Similarly, performance at Year 1 was positively associated with the performance at Year 2. Finally, the lesser the period since formal training, the greater the performance on EXPERTise 2.0 at Year 2, although it constituted a weak predictor, accounting for only 4% of the variance.

## Discussion

The aim of this study was to test the contribution of overall experience, recency of training, perceptions of job excitement, and prior performance on cue utilisation in the context of transmission network power control. Qualified electricity transmission network controllers were tested, one year apart, using the Transmission Network Power Control edition of EXPERTise 2.0. In addition to a series of questions pertaining to their overall experience, recency of training, and perceived level of excitement, the edition comprised four tasks that were designed to test different aspects of cue utilisation as a measure of their capacity for situational assessment.

A hierarchical multiple regression, with years of experience, previous EXPERTise 2.0 performance, recency of training, and perceptions of job excitement entered sequentially as predictor variables, and subsequent performance on EXPERTise 2.0 as the outcome variable, revealed a model with contributions drawn from previous performance and perceptions of job excitement. The full regression model suggested a further contribution of formal training, although a relationship appeared to be evident whereby the recency of formal training was likely to be associated with perceptions of job excitement. No predictive relationship was evident for years of experience.

Consistent with the hypothesis, the outcomes suggest that a significant proportion of the variance in cue utilisation is explained by previous performance. Controlling for job excitement failed to show a contribution of formal training. However, including job excitement into the model revealed a contribution for formal training, suggesting that the latter is likely to constitute an artifact associated with perceptions of job excitement.

The potential relationship between participating in formal training and perceptions of the job as ‘exciting’ suggests that situational assessment, as measured by EXPERTise 2.0, may be sustained in part, by a combination of the recency of formal training underpinned by on-the-job exposure to nonroutine tasks that correspond to perceptions of job excitement. While there is likely to be shared variance that contributes to the cognitive development of operators, it may provide the necessary engagement that sustains situational assessment.

From a theoretical perspective, the lack of a relationship between preceding years of experience and situational assessment might be due to differences in the idiosyncratic nature of operational experience and its contribution to situational assessment. Previous investigations have only revealed a low to moderate relationship between task-related experience and performance on EXPERTise 2.0 (Carrigan et al., 2021; Crane et al., 2018) suggesting that broad measures of experience are imprecise correlates of situational assessment due to the lack of structured exposure to situations that would enable the acquisition and maintenance of cues.

Formal training tends to be structured, and more recent exposure to formal training potentially results in the development and reinforcement of skills and knowledge that are likely to be retained and further developed. This is especially the case where there is corresponding exposure to on-the-job, nonroutine tasks that facilitate the consolidation of previously acquired higher-order skills such as situational assessment.

While the results provide an initial explanation for the maintenance of situational assessment in a highly technical context, other individual differences or system-related issues may constitute an alternative or equally important explanation for the outcomes in Year 2 (Rich et al., 2010). For example, organisational culture may encourage or discourage active involvement in the management of automated systems (Heatherly et al., 2023). Similarly, work design may enable or impede the capacity to engage in nonroutine tasks that contribute to the high-order thinking and sustained situational assessment (Parker & Grote, 2022).

### Applied Implications

Strategies to maintain the capacity for situational assessment are becoming increasingly important in the transition to automated systems where operators retain oversight but otherwise exercise minimal engagement in the management of systems. Although electricity transmission network control constitutes a semi-automated environment, the experience in this context can inform interventions in other high reliability operating environments. These interventions include the need for regular formal training and the creation of an operating environment through job design that enables engagement with nonroutine activities contributing to job excitement (Parker, 2014).

The outcomes of the present research also suggest a need to assess the capacity for situational assessment on a regular basis to identify changes and signal the need for timely interventions. It may also be possible to identify improvements in situational assessment as a consequence of interventions and potentially establish whether operating environments are sufficiently exciting. Finally, operators may be recruited who perceive technical roles as exciting, thereby creating a level of job excitement that contributes to practice and the maintenance of skilled situational assessment.

### Limitations and Future Research

A significant strength and limitation associated with the present research is its sample. The sample comprised highly experienced electricity transmission network controllers rather than naïve participants. This limited population translated into a limited sample. While the effects were strong, there are significant limitations associated with the use of multiple regression for small samples. Future research would clearly benefit from replication with a larger sample and in a different domain to establish whether the effects are sufficiently robust across different editions of EXPERTise 2.0. It would also provide a degree of confidence in further establishing the reliability of the EXPERTise 2.0 tasks.

While Pearson’s correlations failed to reveal any relationships between the predictor variables, Bayes Factor correlations provided what might be interpreted as substantial support for a relationship, particularly between the recency of formal training and perceptions of job excitement. This suggests an opportunity in future research to consider the relationship between opportunities for formal training and perceptions of job excitement, particularly in the context of advanced technology operating environments. In the present study, perceptions of job excitement were constrained to a single question and there is an opportunity to consider allied constructs, including exposure to nonroutine activities, and job motivation.

Although the construct validity of EXPERTise 2.0 has been established in electricity control, an opportunity exists to consider changes in performance over an extended period and identify the rate at which situational assessment decays beyond changes in performance on EXPERTise 2.0. For example, changes in interface design and/or procedures will interact with the capacity for situational assessment. Establishing the nature of these relationships will improve the capacity for risk assessment and improve organisational decision making.

### Conclusion

The aim of this study was to determine the features that contribute to sustained situational assessment in the context of transmission network power control. Participants were tested using the Transmission Network Power Control edition of EXPERTise 2.0 on two occasions, one year apart. The results revealed a statistically significant model with the variance in Year 2 explained largely by a combination of performance in Year 1, the recency of formal training, and the level of perceived excitement associated with the role. Experience in network control was not associated with situational assessment performance in Year 2.

The results highlight the benefit in testing situational assessment on a regular basis, particularly during the introduction of advanced technology such as automation. They also suggest that any deterioration in situational assessment may be mitigated by ensuring that the role carries with it a degree of excitement for operators.

## Key Points

• Electricity controllers require situational assessment to ensure performance
• Sustaining situational assessment skills can be challenging.
• Situational assessment skills may be sustained by a combination of previous performance, a perception of the role as exciting, and more recent training.
